# Artificial intelligence for reduced dose 18F-FDG PET examinations: a real-world deployment through a standardized framework and business case assessment

**DOI:** 10.1186/s40658-021-00374-7

**Published:** 2021-03-09

**Authors:** Katia Katsari, Daniele Penna, Vincenzo Arena, Giulia Polverari, Annarita Ianniello, Domenico Italiano, Rolando Milani, Alessandro Roncacci, Rowland O. Illing, Ettore Pelosi

**Affiliations:** 1 Affidea BV, Amsterdam, Netherlands; 2 PET Center, Affidea IRMET, Torino, Italy; 3 Affidea, Budapest, Hungary; 4 University College London United Kingdom, London, UK

**Keywords:** Artificial intelligence, PET/CT, Dose reduction, Image interpretation

## Abstract

**Background:**

To determine whether artificial intelligence (AI) processed PET/CT images of reduced by one-third of 18-F-FDG activity compared to the standard injected dose, were non-inferior to native scans and if so to assess the potential impact of commercialization.

**Materials and methods:**

SubtlePET™ AI was introduced in a PET/CT center in Italy. Eligible patients referred for 18F-FDG PET/CT were prospectively enrolled. Administered 18F-FDG was reduced to two-thirds of standard dose. Patients underwent one low-dose CT and two sequential PET scans; “PET-processed” with reduced dose and standard acquisition time, and “PET-native” with an elapsed time to simulate standard acquisition time and dose. PET-processed images were reconstructed using SubtlePET™. PET-native images were defined as the standard of reference. The datasets were anonymized and independently evaluated in random order by four blinded readers. The evaluation included subjective image quality (IQ) assessment, lesion detectability, and assessment of business benefits.

**Results:**

From February to April 2020, 61 patients were prospectively enrolled. Subjective IQ was not significantly different between datasets (4.62±0.23, *p*=0.237) for all scanner models, with “almost perfect” inter-reader agreement. There was no significant difference between datasets in lesions’ detectability, target lesion mean SUV_max_ value, and liver mean SUV_mean_ value (182.75/181.75 [SD:0.71], 9.8/11.4 [SD:1.13], 2.1/1.9 [SD:0.14] respectively). No false-positive lesions were reported in PET-processed examinations. Agreed SubtlePET™ price per examination was 15-20% of FDG savings.

**Conclusion:**

This is the first real-world study to demonstrate the non-inferiority of AI processed 18F-FDG PET/CT examinations obtained with 66% standard dose and a methodology to define the AI solution price.

**Supplementary Information:**

The online version contains supplementary material available at 10.1186/s40658-021-00374-7.

## Introduction

Positron emission computed tomography/computed tomography (PET/CT) is widely used in various clinical applications, including the investigation of oncological and neurological disorders [1]. The evolution of technology and the extended clinical applications of PET/CT have led to a notable worldwide increase of the number of scans performed [2]. Due to the leading role of PET/CT in the evaluation of systemic therapy response, a significant number of patients undergo more than one PET/CT scan per year, thus increasing patients’ radiation exposure. Radiation dose has been associated with a slight increase in patients’ lifetime risk of developing cancer [3]. Legal framework, such as EURATOM 2013/59 Directive [4], has been published for the optimization of patients’ radiation exposure from imaging practices.

Recently, reconstruction algorithms have been developed to improve the quality of images acquired with reduced administered radiotracer. However, these procedures are complicated, time-consuming and do not produce satisfactory outcomes when the injected activity is significantly lower compared to the standard dose suggested by the procedure guidelines [5]. Machine learning methods have been developed to resolve these issues [6] by utilizing paired low-dose and standard-dose images to train models that can predict standard-dose images from low-dose inputs [7].

**Fig. 1:**
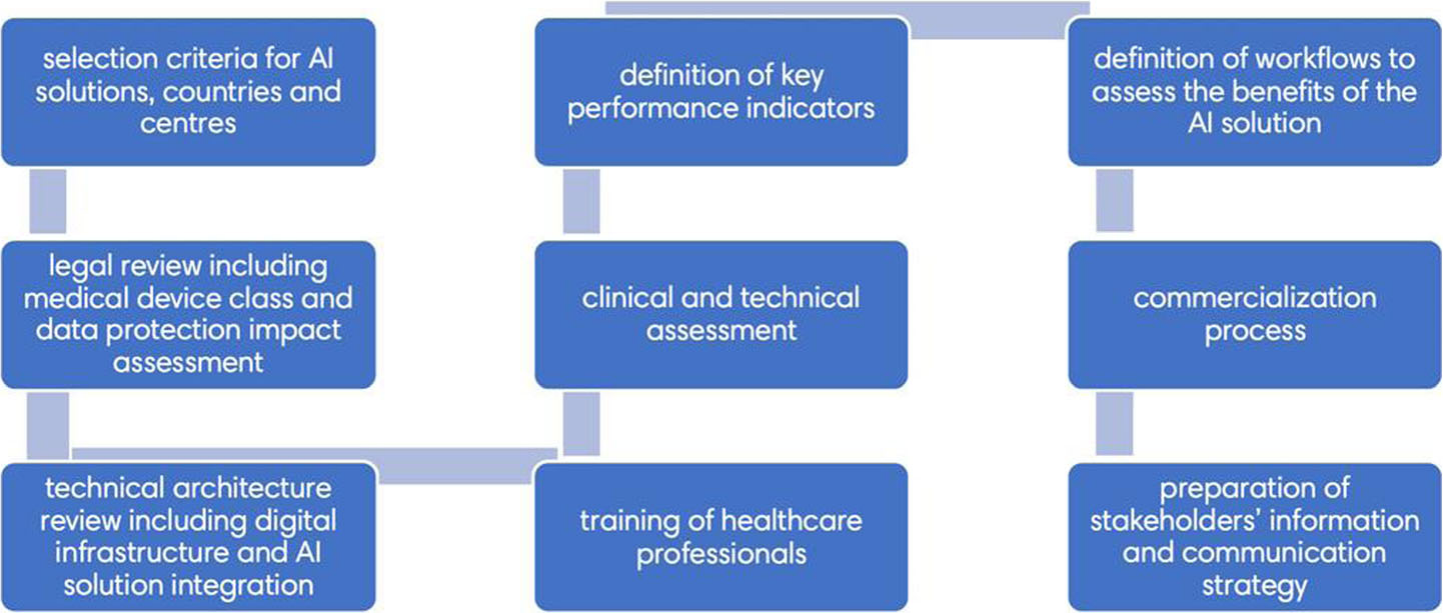
Nine-stage framework for AI deployment in clinical practice

The value proposition of artificial intelligence (AI) in healthcare has been well described [8]; however, real-world use in clinical practice remains limited [9]. A multinational healthcare organization developed a nine-stage framework (Fig. 1) to deploy AI solutions into its network. The framework was designed to allow objective assessment of where the clinical and business benefits lie [10, 11]. Using this framework, a machine learning solution enabling reduced administered radiotracer activity PET/CT scans was introduced into a single center in Italy. The purpose of this study was to determine whether it was able to produce images of adequate diagnostic confidence which were considered non-inferior to native scans with two different PET/CT scanner models, and if so to assess the potential impact of commercialization to the business.

## Materials and methods

**Fig. 2:**
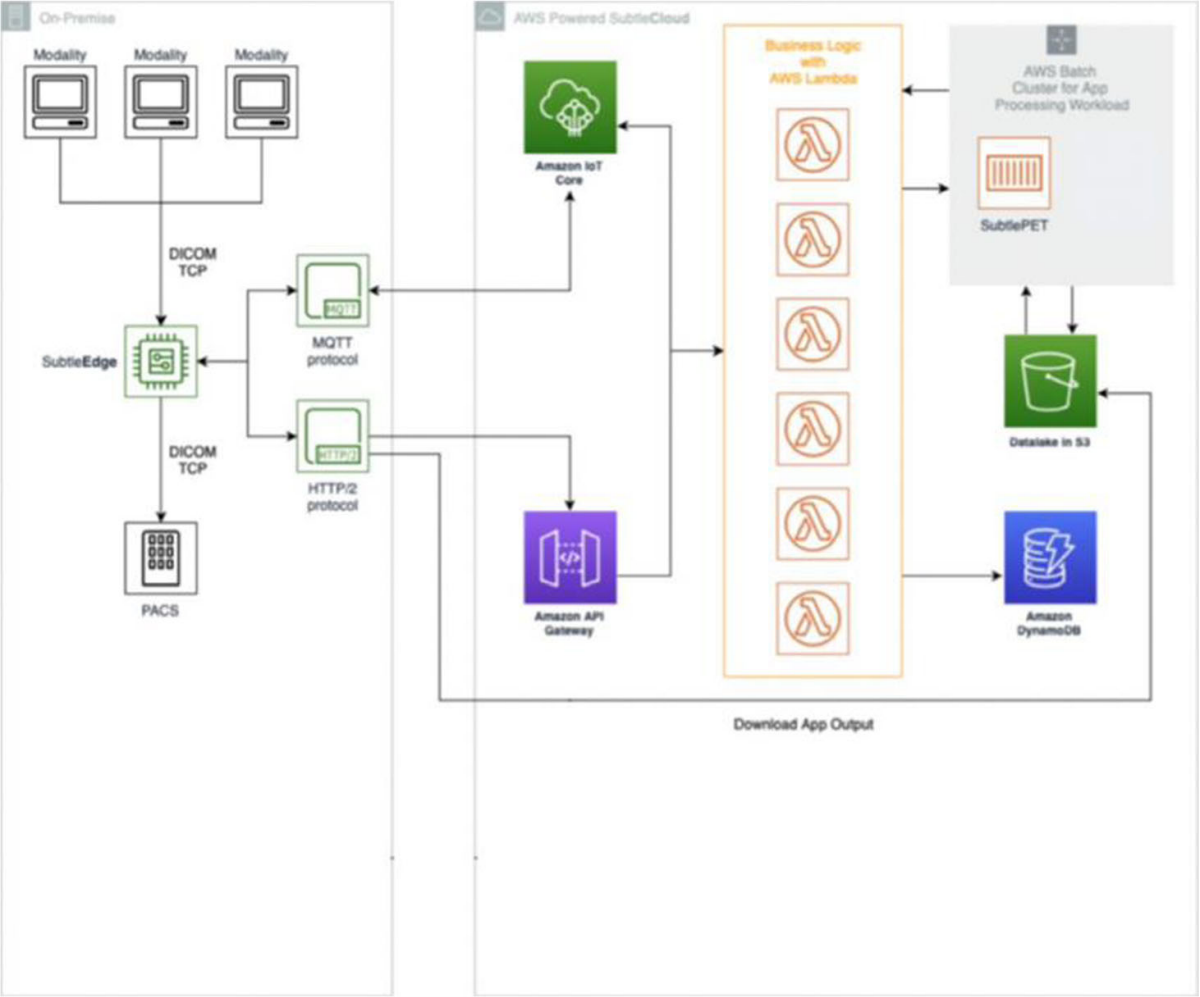
Technical integration architecture and communication

According to the framework, the SubtlePET™ AI solution (Subtle Medical, Menlo Park, CA, USA) was selected to be assessed in a single, European Association of Nuclear Medicine (EANM) Research Ltd. (EARL) [12] accredited, PET/CT center with three scanners. Appropriate legal review was undertaken to ensure that the solution was certified with the correct Medical Device classification and was compliant with the European General Data Protection Regulations (GDPR). In parallel, the technical architecture (Fig. 2) for the integration of the solution was reviewed. Once the legal and technical requirements were validated, the software was installed, configured, and verified, and the personnel were trained on its use and limitations. Deep learning processing of the low dose data was performed offline using an FDA-cleared convolutional neural network (CNN) based, deep learning, AI image enhancement software product, SubtlePET™ (Version 1.3, Subtle Medical, Menlo Park, CA). SubtlePET^TM^ was trained on hundreds of multi-vendor PET and PET-CT datasets featuring a range of image quality. Deep learning processing time per series is typically between 1-2 min. SubtlePET™ software uses a convolution neural network-based algorithm to reduce noise and improve image quality of fluorodeoxyglucose (FDG) and amyloid PET and PET/CT images [6, 13]. Even though SubtlePET™ is certified and validated for clinical use for all major PET/CT vendors and many different models, according to the framework, a clinical assessment had to be undertaken. This prospective analysis was designed to verify the performance of the algorithm using real-world data.

### Patient enrollment

Patients referred for 18F-FDG PET/CT during diagnostic work-up for oncological disease were screened for prospective enrollment. Inclusion criteria were (a) age >18 years; (b) FDG-avid malignancy; (c) glycemia <180 mg/dL; (d) adequate physical condition to allow them to remain still for approximately 40 min, for two consecutive PET scans. Claustrophobic patients were excluded. Patients meeting these criteria were approached to participate in the study. According to GDPR and institutional procedures related to the information provided to patients for the examination process, all the patients signed an informed consent form prior to any study procedures.

### Examination protocol

PET images were acquired with three different 3D PET/CT scanners (Discovery ST-4—PET scanner 1, Discovery ST-16—PET scanner 2, and Discovery IQ—PET scanner 3) from the same manufacturer (GE Healthcare, Milwaukee, WI, United States) without time of flight (TOF) technology. 18F-FDG was provided by Advanced Accelerator Applications pharmaceuticals (AAA by Novartis, Saint-Genis-Pouilly, France) in compliance with Good Manufacturing Practice (GMP) and in accordance with EANM procedure guidelines [5].

For the purposes of this study, FDG doses were reduced by one-third compared to the standard injected dose to a patient with the same body weight, according to institutional procedure guidelines. All doses were injected via peripheral venous catheter.

During the same day, patients underwent two sequential PET scans in continuous-bed-mode: a reduced dose acquisition scan (PET-processed) and a reference acquisition scan (PET-native). The PET-processed scan was acquired first at 60 min post-injection from skull base to mid-thigh. To simulate normal acquisition time and reduced injected dose, PET images for scanners 1 and 2 were acquired at 2.5 min per bed-position, while for scanner 3, images were acquired at 1.5 min per-bed position, in accordance with institutional procedure guidelines. Following the PET-processed scan, the PET-native scan was acquired for the same region without moving the patient. The PET-native images were acquired with an elapsed time, increasing the minutes per bed-position, to simulate normal acquisition time and standard injected dose. To define the PET emission acquisition time that simulated a full dose examination, a phantom study was performed on each PET/CT scanner using cancer imaging conditions, applying the following equation: standard time acquisition per bed × exp(900×λ) × 1.25 (s).

Patients underwent one low-dose CT prior to PET-processed acquisition, for attenuation correction and anatomical correlation of PET findings. Emission data was corrected for randoms, dead time, scatter, and attenuation and was reconstructed iteratively by an ordered-subsets expectation maximization (OSEM) algorithm. Three different PET/CT scanners were utilized: Discovery ST-4, Discovery ST-16, and Discovery IQ. The Discovery-ST (GE Medical Systems) combines a helical multi-slice CT scanner (respectively 4 and 16 slices) and a BGO block detector PET tomograph. The Discovery ST has good overall performances in 3D acquisition mode, with balanced performance of the several physical parameters influencing the final image quality. In particular, high sensitivity results in low statistical noise and the consequent recovery of spatial resolution in clinical studies. Furthermore, the good NEC response allows clinical studies to be optimized in terms of the signal to noise ratio. The Discovery-IQ combines a BGO-based PET tomograph with a 16-slice CT scanner; the software includes the algorithm VUE-point HD (VPHD). The Discovery-IQ PET/CT scanner with 5-ring detector blocks has the highest overall performance of the Discovery BGO-based scanners, with further improvement in sensitivity and counting rate performance. According to institutional processes, images were reviewed for artifacts by the technologist before the patient was discharged. Upon confirmation, PET-processed acquisitions were sent by the radiographer from the modality to the Subtle server (SubtleEdge) for processing. Incoming images were automatically anonymized and quality controlled (QC) according to the SubtlePET™ process. Images that passed QC were processed and were sent automatically to the Picture Archiving and Communication System (PACS) in an average time of 10 min.

### Image quality assessment

The PET-native images were defined as the standard of reference and were reviewed by two independent physicians who had access to all the clinical, imaging, and reconstruction data, to reach to consensus report that was delivered to the patient within 24 h.

The PET-processed and PET-native datasets were anonymized, separated, and randomized allowing independent assessment of each dataset over a 4-week period, by four blinded board-certified nuclear medicine physicians, with more than 5 years’ experience (EP and VA> 15 years; GP and AI > 5 years). Each reviewer assessed all datasets. They were blinded regarding image acquisition, reconstruction technique, and clinical information. 18F-FDG PET/CT images were reported according to EANM procedure guidelines [5].

For image quality, the PET datasets were rated on a 5-point scale (1: very poor/non-diagnostic; 2: poor; 3: moderate; 4: good; and 5: excellent) with scores 4 and 5 considered adequate to provide diagnostic confidence.

Furthermore, each reviewer had to give their opinion as the whether they were reviewing the PET-processed or the PET-native dataset or if this was indeterminate.

Lastly, the detectability of all lesions was evaluated in a per-lesion analysis. In patients with ten lesions or fewer, all lesions were assessed by the reviewers, while in patients with more than ten lesions, those ten with the highest standard uptake values (SUV) max were included in the analysis. In the two datasets, the SUV_max_ of the largest lesion and the SUV_mean_ of the liver were measured. SUV was defined as activity concentration (Bq/mL) divided by injected activity (Bq) normalized to body weight. The highest voxel value (SUV_max_) and the mean voxel value (SUV_mean_) were obtained in a volume of interest (VOI) covering the entire tumor as defined by each reviewer. Considering that all PET-native images were acquired after the PET-processed images, a correction factor for the SUV values was calculated according to Appendix 1 and Supplemental Table 1 [14]. Lesions not detected by a reviewer in a specific dataset were assigned as 0.

Once the independent analysis of the native and processed datasets was complete, they were logged and unblinded. The whole dataset was scrutinized to determine whether the processed scans were non-inferior to the native scans. Quantitative assessment was of lesion detectability and SUV levels; qualitative assessment on subjective image quality. Inter-observer variability for image quality assessment was performed. Differences in results between PET/CT scanner models were also assessed.

### Statistical analysis

Descriptive statistics for categorical variables were presented as relative/absolute frequencies, while those for continuous ones as the median (range). The inferential analyses for categorical and continuous variables were performed by the Fisher’s exact test and the Mann–Whitney test, respectively. The degree of agreement among reviewers for evaluating image quality was assessed using intraclass correlation coefficients (ICC) and their 95% CI, using a 2-way mixed, single measure, consistency model. ICC was interpreted according to Landis J. R. interpretation scale [15] (0.0: poor; 0.0-0.20: slight; 0.21-0.40: fair; 0.41-0.60: moderate; 0.61-0.80: substantial; 0.81-1.00: almost-perfect reproducibility). To analyze the lesion detectability in the two PET datasets, the detection rate was calculated for each reviewer based on the total number of suspected lesions determined by the standard of reference. All *p* values were obtained by the two-sided exact method at the conventional 5% significance level. Data were analyzed with R 3.6.1 (R Foundation for Statistical Computing, Vienna-A, http://www.R-project.org). Once the analysis of the outcomes from the clinical evaluation was completed, an assessment of the business benefits was performed. The potential net savings from the use of SubtlePET™ were calculated using data from the whole PET/CT network, not just the single center, assuming replicability of results. A percentage of these savings was then agreed as a fair price for the AI solution.

## Results

From February to April 2020, 1167 patients were referred for an 18F-FDG PET/CT examination at our facility. From the total referred patients, 107 (9.2%) complied to the eligibility criteria, out of which 46 did not consent to participate in the study. Sixty-one patients, 36 (59.1%) female and 25 (40.9%) males were prospectively enrolled to the study. The median age was 66 years (33-84 years), median weight was 67 kg (40-100 kg) and the tumor sub-types investigated were ten. Patients were injected with a median dose of 131 MBq (99-199 MBq) of 18F-FDG; 66% of the median standard institutional dose of 196 MBq (148-296 MBq). Twenty examinations were performed with PET scanner 1, 21 with PET scanner 2 and 20 with PET scanner 3. The mean time interval between tracer injection and the second PET scan time point was 75 min for PET scanner 1 and 80 min for PET scanners 2 and 3. Data is presented in Table 1 and Supplemental Table 2.

**Table 1:** Population characteristics

**Patient characteristics**	**Number (%) [*N*** **= 61]**
Male	25 (40.9%)
Female	36 (59.1%)
PET scanner 1 (GE Discovery ST-4)	20 (32.8%)
PET scanner 2 (GE Discovery ST-16)	21 (34.4%)
PET scanner 3 (GE Discovery IQ)	20 (32.8%)
**Tumor characteristics**	
Rectal-colon cancer	13 (21.3%)
Lung cancer	13 (21.3%)
Hodgkin/non-Hodgkin lymphoma	9 (14.8%)
Gynecological cancer	8 (13.1%)
Breast cancer	8 (13.1%)
Melanoma	4 (6.6%)
Pancreatic cancer	2 (3.3%)
Gastric cancer	2 (3.3%)
Cholangiocarcinoma	1 (1.6%)
Head and neck cancer	1 (1.6%)
	**Median (range)**
Age	66 years (33-84)
Body weight	67 kg (40-100)
Dose injected (study protocol)	131 MBq (99-199)
Reference dose (standard procedure)^a^	196 MBq (148-296)

^a^These values represent the routine administered dose of 18F-FDG according to the institutional standard of care procedures

### Qualitative image quality assessment

**Fig. 3:**
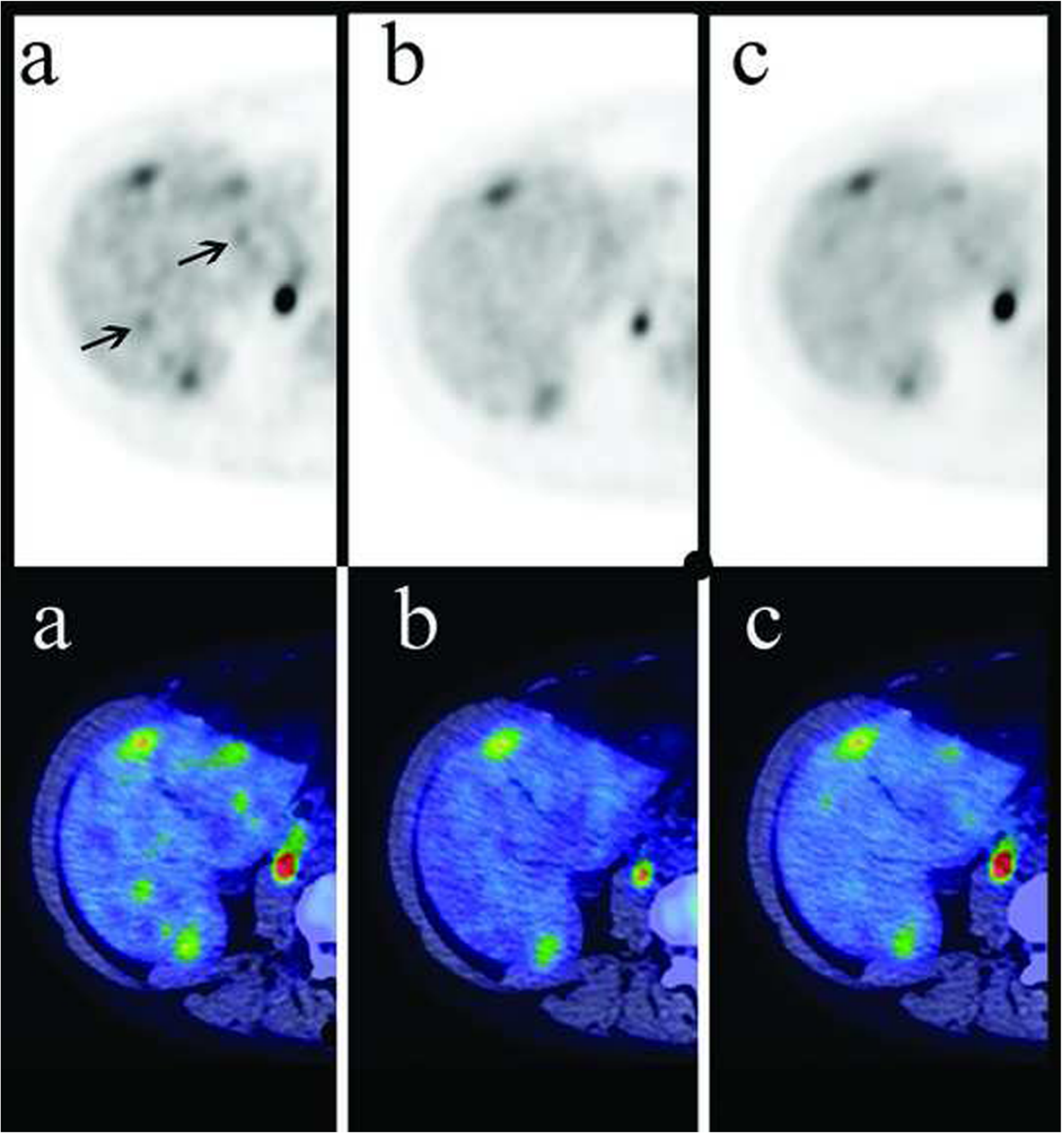
18F-FDG liver biodistribution in a patient affected by liver metastases and referred to PET/CT to restage the disease after systemic therapy. PET and Fusion images were evaluated applying the same SUV_max_ threshold. **a** Scan with reduced number of counts and standard acquisition (no processed image): highly inhomogeneous radiotracer uptake showing multiple focal uptakes that might be interpreted as metastases and thus, erroneously, as progressive disease. **b** Native scan (acquisition with increased time): regular distribution of the radiotracer leading to the correct identification of few metastatic lesions (scan correctly reported as stable disease). **c** Processed scan, obtained with reduced dose and processed by SubtlePET: regular radiotracer distribution in the liver, confirming the non-inferior quality of the image compared to native scan. The interpretation of these image lead to the same conclusion derived by the interpretation of the native scan (stable disease)

As shown in Fig. 3, the low-dose non-processed image presents with high inhomogeneous liver uptake and lead to wrong interpretation of the scan.

**Fig. 4:**
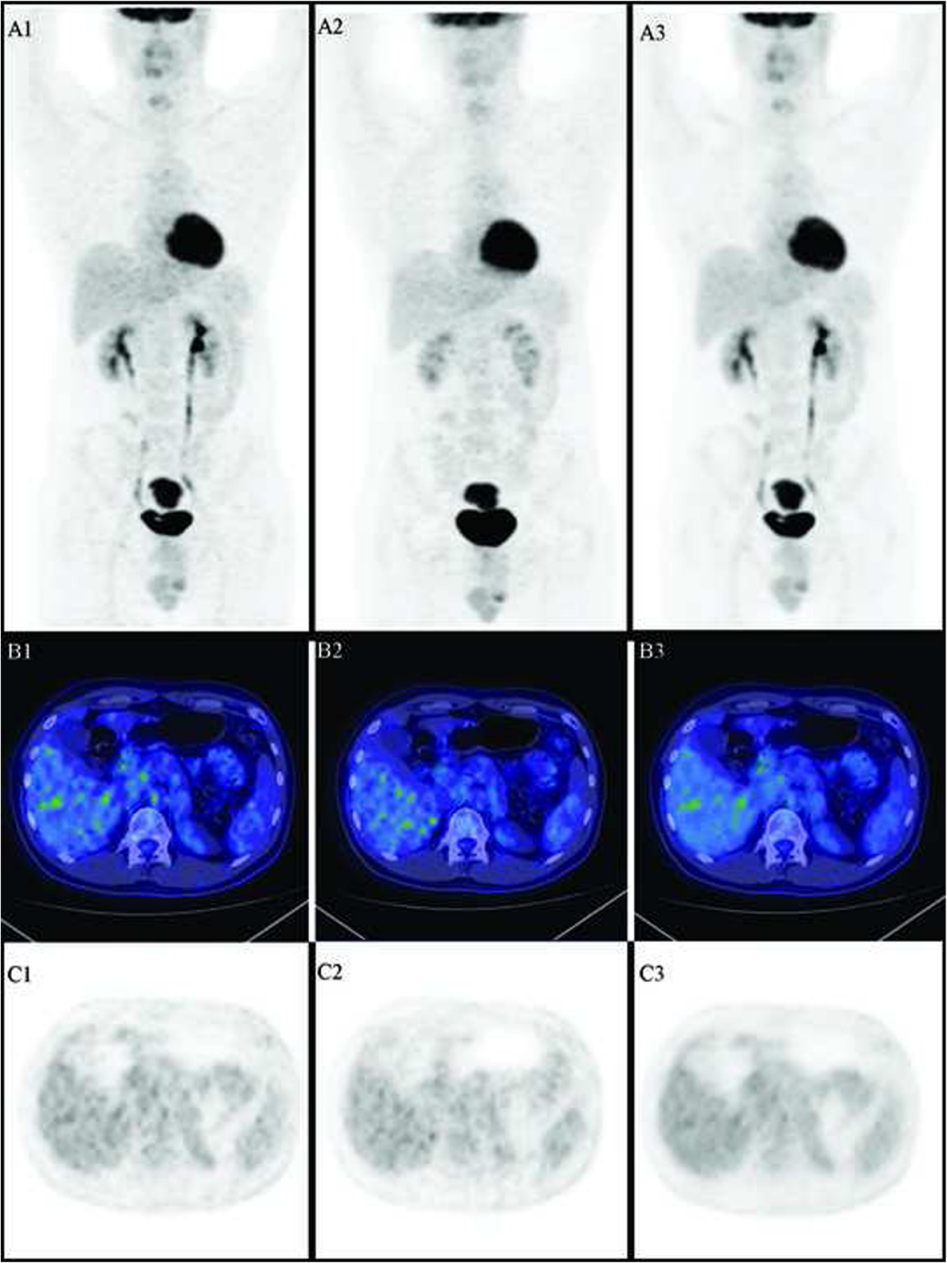
Staging of sigmo-rectal adenocarcinoma (new diagnosis). Patient injected with 66% dose of 18F-FDG. 1. Scan with reduced number of counts (acquisition with standard time and 66% FDG dose). 2. Native scan: scan with standard number of counts (acquisition with increased time). 3. Processed scan, resulting from the study with reduced counts (1) processed by AI SubtlePET^TM^. **a** Maximum intensity projection (MIP). **b** Fused transaxial positron emission tomography and computed tomography. **c** Transaxial positron emission tomography images. All images were visualized with AW server station by setting the same parameters (DFOV 52.0 × 32.2 cm, *M*=4.49 g/ml). Distribution of the radiotracer, in the MIP and in the transaxial images at the level of the liver, shows a good image quality both in native and in the processed study

**Fig. 5:**
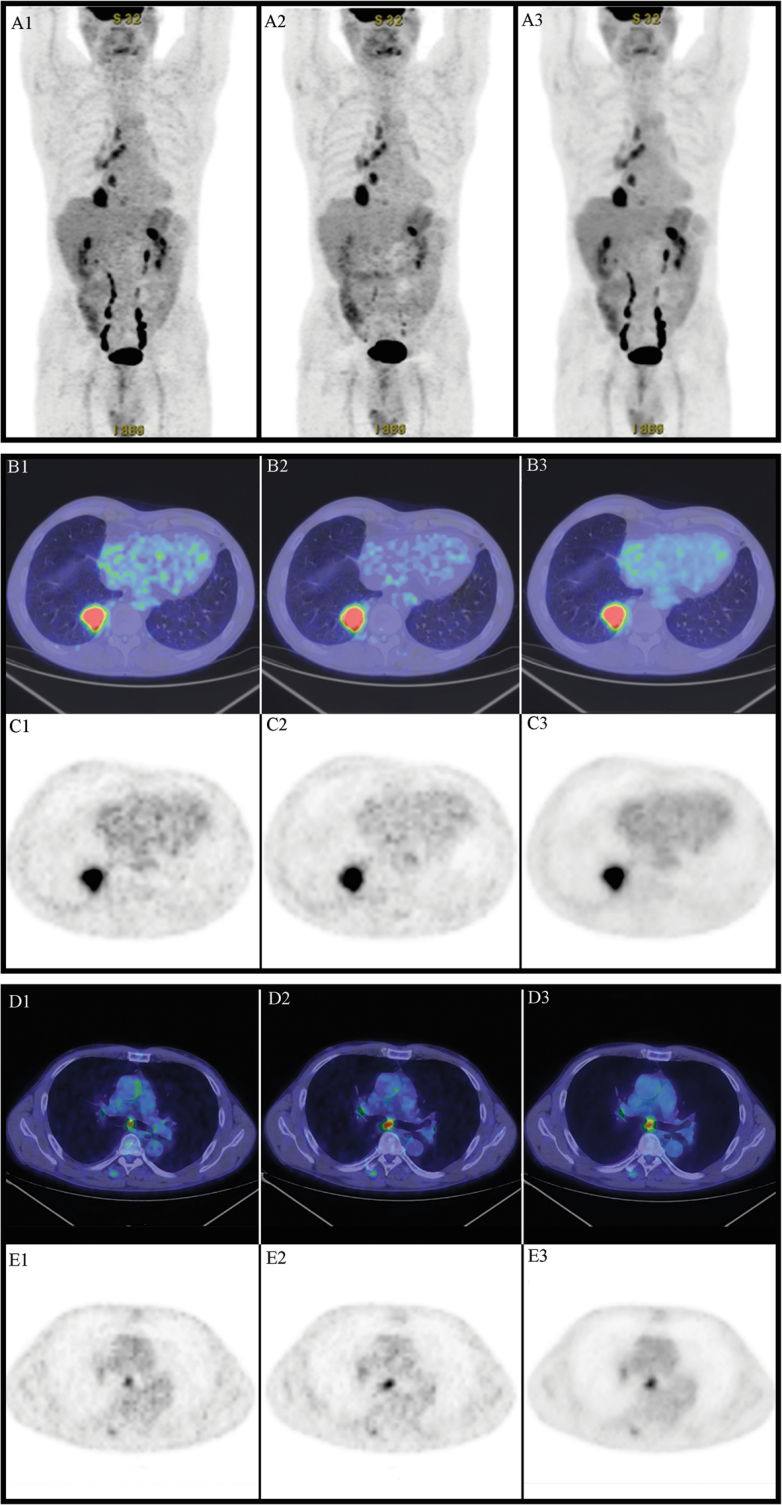
Metabolic characterization of a right lung nodule. Patient injected with 66% dose of 18F-FDG. 1. Scan with reduced number of counts (acquisition with standard time and 66% FDG dose). 2. Native scan: scan with standard number of counts (acquisition with increased time). 3. Processed scan, resulting from the study with reduced counts (1) processed by AI SubtlePET^TM^. **a** Maximum Intensity Projection (MIP). **b**, **d** Fused transaxial positron emission tomography and computed tomography images. **c**, **e** Transaxial positron emission tomography images. All images were visualized with AW server station by setting the same parameters (DFOV 57.8 × 35.8 cm, *M*=6.01 g/ml). Lung lesion is easily and appropriately characterized by both native and AI SubtlePET^TM^ images, see MIP (Fig. 5a) and transaxial (Fig. 5b); suspected metastatic lymph nodes are well visualized in both native and AI SubtlePET MIP (Fig. 5a) and transaxial images (Fig. 5c)

**Fig. 6:**
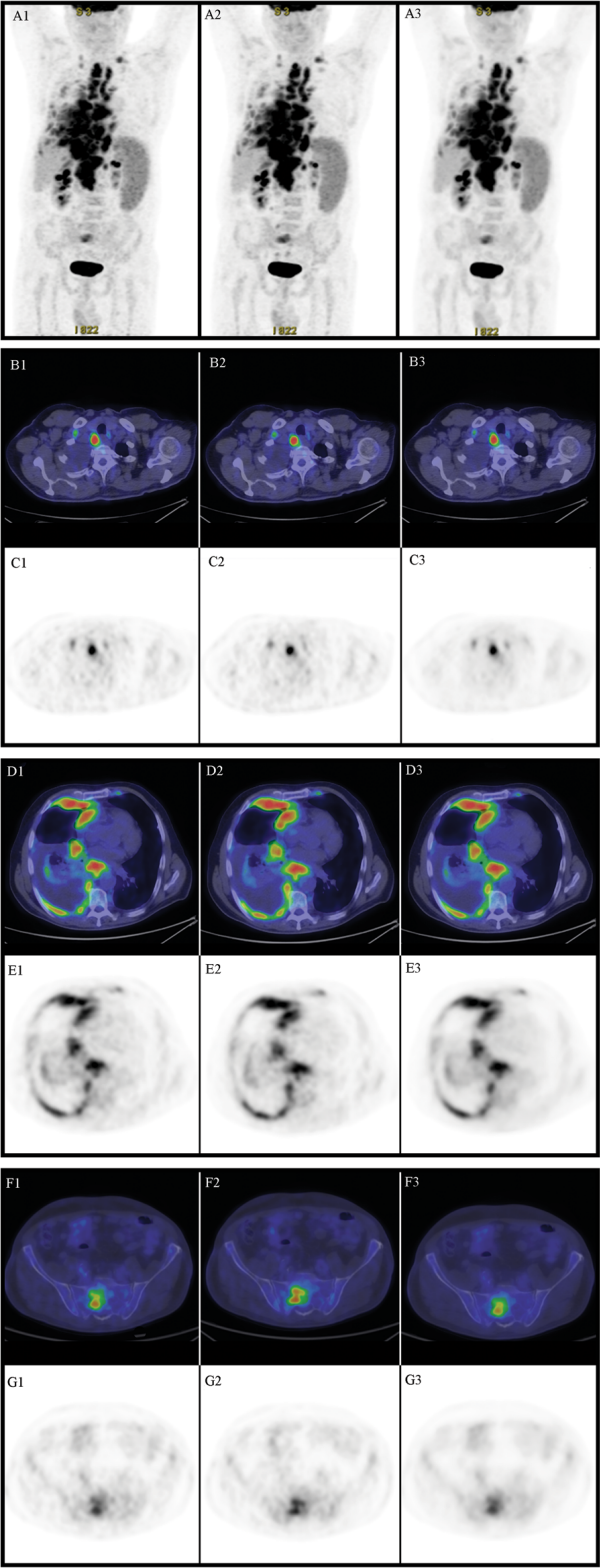
Staging in patient with pleural recurrence of mantle cell lymphoma. 1. Scan with reduced number of counts (acquisition with standard time and 66% FDG dose). 2. Native scan: scan with standard number of counts (acquisition with increased time). 3. Processed scan, resulting from the study with reduced counts (1) processed by AI SubtlePET^TM^. **a** Maximum intensity projection (MIP). **b**, **d**, **f** Fused transaxial positron emission tomography and computed tomography. **c**, **e**, **g** Transaxial positron emission tomography images. All images were visualized with AW server station by setting the same parameters (DFOV 57.8 × 35.8 cm, *M*=6.31 g/ml). The comparison between the studies shows a good quality of the processed by AI SubtlePET^TM^ images in the MIP visualization (Fig. 6a) and in the transaxial images, identifying the disease at the level of lymph nodes in the upper chest (Fig. 6b), at the level of the thorax (Fig. 6c) and at the sacrum (Fig. 6d)

Vice versa, image quality had a mean score of 4.62 ± 0.23 for the PET-processed dataset and 4.54 ± 0.20 for the PET-native dataset, a difference that was not statistically significant (*p*=0.237). Figures 4, 5, and 6 represent some examples of the image quality. Results of the statistical analysis are presented in Table 2.

**Table 2:** Image quality assessment using a 5-point scale. The analysis was performed considering the mean score of the four reviewers and the score assigned by each individual reviewer

	PET-processed	PET-native	
	Mean ± SD (range)	Mean ± SD (range)	*p*
Overall (mean score of the 4 reviewers)	4.62 *± 0.23* (*1-5*)	4.54 *± 0.20* (*2-5*)	0.11
Reviewer 1 (AI)	4.73 *± 0.44* (*4-5*)	4.65 *± 0.48* (*4-5*)	
Reviewer 2 (EP)	4.55 *± 0.59* (*3-5*)	4.38 *± 0.66* (*3-5*)	
Reviewer 3 (GP)	4.29 *± 1.01* (*1-5*)	4.31 *± 0.65* (*2-5*)	
Reviewer 4 (VA)	4.90 *± 0.35* (*3-5*)	4.80 *± 0.48 (3-5*)	

The degree of agreement among reviewers for the image quality evaluation was classified as “almost-perfect” for both PET-processed and PET-native images, with an ICC of 0.982 (95% CT=0.793-0.934) and 0.921 (95% CI= 0.855-0.943) respectively.

PET processed images and PET-native images were easily and properly identified with almost perfect accuracy by all the four readers. Namely, PET-processed images in 97.9% of cases, and the PET-native images in 97.2% of cases.

### Quantitative image quality assessment

According to the standard of reference, 183 lesions were detected in 46 examinations. In 15 examinations, no lesions were detected. The mean number of lesions detected by the four reviewers was 182.75 ± 3.1 for the PET-processed examinations and 181.75 ± 2.8 for the PET-native images. No reviewer reported false-positive lesions in the PET-processed examinations. Results are presented in Table 3.

**Table 3:** The analysis was performed considering the mean lesions detected by the four reviewers and by each individual reviewer

Lesions detectability for PET-processed and PET-native datasets			
	PET-processed	PET-native	
	Number of lesions	Number of lesions	*p*
Overall (mean score of the 4 reviewers)	182.8 ± 3.1	181.8 ± 2.8	0.22
Reviewer 1 (AI)	185	184	
Reviewer 2 (EP)	186	183	
Reviewer 3 (GP)	178	177	
Reviewer 4 (VA)	182	183	

A mean number of 2.98 ± 0.05 lesions per patient were detected by all reviewers in PET-processed images and 2.99 ± 0.05 in PET-native images. Results are presented in Table 4.

**Table 4:** The analysis was performed considering the mean lesions detected by the four reviewers and by each individual reviewer

Number of lesions detected per patient for PET-processed and PET-native datasets			
	PET-processed	PET-native	
	Number of lesions	Number of lesions	*p*
Overall (mean score of the 4 reviewers)	2.98 ± 0.05	2.99 ± 0.05	0.51
Reviewer 1 (AI)	3.01	3.03	
Reviewer 2 (EP)	3.00	3.04	
Reviewer 3 (GP)	2.90	2.91	
Reviewer 4 (VA)	3.00	2.98	

The mean SUV_max_ value measured in the target lesions (lesion with highest SUV_max_) was 9.8 ± 8.8 in the PET-processed dataset and 11.4 ± 9.8 in the PET-native dataset. The mean SUV_mean_ value of the liver was 2.1 ± 0.7 in the PET-processed dataset and 1.9 ± 0.7 in the PET-native dataset. The difference was not statistically significant in either (all *p*>0.05).

### PET scanners reproducibility

The analysis of the evaluation of image quality and lesion detectability was performed in three sub-cohorts, stratifying the overall population by the three PET scanner models. Both the quantitative and qualitative results did not produce any significant differences between the scanner models and are presented in Table 5.

**Table 5:** Image quality assessment, lesion detectability, and SUV values for three different PET scanner models

Comparison between three different PET scanner models						
PET scanner	Dataset	Dataset identification	Image quality (1 to 5)	Number of lesions	SUV_**max**_ of target lesion	SUV_**mean**_ of the liver
			Mean ± SD	Mean ± SD	Mean ± SD	Mean ± SD
**1 (*n*=20)**	PET-native	96.2%	4.55 ± 0.61	1.91 ± 3.07	15.80 ± 11.64	1.87 ± 0.84
	PET-processed	97.5%	4.57 ± 0.76	1.91 ± 3.07	13.84 ± 9.89	1.99 ± 0.86
**2 (*n*=21)**	PET-native	98.8%	4.54 ± 0.59	3.58 ± 3.48	13.21 ± 10.8	1.81 ± 0.37
	PET-processed	100%	4.70 ± 0.51	3.55 ± 3.50	11.35 ± 9.96	2.00 ± 0.46
**3 (*n*=20)**	PET-native	96.2%	4.53 ± 0.62	3.53 ± 3.63	7.07 ± 4.63	2.19 ± 0.77
	PET-processed	96.2%	4.60 ± 0.74	3.52 ± 3.65	6.05 ± 4.39	2.36 ± 0.81

*PET scanner 1*= Discovery ST-4; *PET scanner 2*= Discovery ST-16; *PET scanner 3*= Discovery IQ

### Business model calculation

The total number of F18-FDG examinations performed in the facility in 2019 and the respective cost of the ordered radiopharmaceutical were used to calculate the potential net savings from the introduction of SubtlePET™. The business model projected 25% savings instead of 33%, to account for a possible increase in the radiopharmaceutical price from the supplier due to the reduction of the total amount ordered. The annual cost of the use of SubtlePET™ agreed by both parties was 15-20% of the gross annual radiopharmaceutical savings.

## Discussion

This is the first real-world study to assess the clinical use of AI for dose reduced 18F-FDG PET/CT examinations and methodology to define a pricing model.

A single center study on the feasibility of 18F-FDG dose reduction in PET/MR examinations was recently published, in which imaging of reduced doses was simulated by reconstruction with different percentages of the original 20 patient’s data [16]. The study concluded that the detection rate and semi-quantitative analysis results were not affected by 50% dose reduction in 18F-FDG PET/MR 6 min/bed whole body examinations. The key difference between the two studies was that we used real data instead of simulated data reducing the administered dose to 66% of standard and also focused on 18F-FDG PET/CT, using a greater number of patients and systems, blinded reviewers, and including details of a business case.

In our study, the decision to reduce the administered dose of 18F-FDG by 33% was based on the assumption that the prolonged duration of the examination for two consecutive PET scans, would be less likely to lead to patient movement artifacts and more likely to be proved non-inferior than the previously described simulations reducing activity by 50%. Although the reviewers could identify whether they were reviewing the PET-processed or PET-native datasets in almost 98% of the cases, the mean image quality score of the datasets was not significantly different and there was “almost-perfect” agreement among reviewers. There was no significant difference in the mean number of lesions detected by the four reviewers compared to the reference standard, with no reviewer reporting false-positive lesions in the PET-processed examinations. There were also no significant statistical differences in the mean number of lesions detected per patient, mean SUV_max_ value measured in the target lesions, and the mean SUV_mean_ value of the liver. Therefore, the AI-processed, 33% dose reduced images were deemed to be “non-inferior” to native images.

In addition, there were no significant differences in the subjective image quality of the datasets and number of lesions detected between the different PET scanners.

Based upon the equivalence of processed and native images, a business case was defined in which the cost of the algorithm was determined to be between 15 and 20% the overall cost saving in 18F-FDG ordered. This commercial arrangement was agreed between both AI developer and service provider for the network, with differing AI cost per scan based upon local 18F-FDG pricing and examinations volume.

Ideally, a direct comparison of dose-reduced/processed images would be made to native images obtained from a scan performed according to the routine clinical protocol. A limitation of the study was that only one dose of 18F-FDG could be administered due to radiation protection limitations. This meant that the native images had to be acquired sequentially with a lower administered dose but longer acquisition times. A correction factor was used to account for this but may have affected the quality of the native images acquired therefore minimizing the differences between image quality. A further limitation was that the pathology was not controlled for, with ten different cancer types included in the 61-patient cohort. Although different pathological entities have differing avidities to 18F-FDG, the methodology chosen should have controlled for this variability. Although the reviewers were blinded, they were still able to accurately determine whether the scans were processed or native. This presents a potential bias, should the reviewers decide to assign a more favorable or worse image quality to the images they know are processed, depending on their own prejudice regarding artificial intelligence.

## Conclusion

To our knowledge, this study provides the first “real world” data comparing actual dose-reduced, AI processed images with native scans, rather than simulation. Given the robust methodology involving a large patient cohort, multiple blinded reviewers, and three different systems, we can be confident that for adult patients undergoing 18F-FDG PET, reducing the dose to 66% of standard produces images with non-inferior image quality when processed by SubtlePET^TM^ in a reproducible manner. Further studies should be undertaken to determine the lower limit of 18F-FDG administration that AI processing will allow while still preserving diagnostic image quality, and whether these results are reproducible in pediatric population in which dose reduction is even more pertinent.

## Supplementary Information

**Additional file 1: Appendix 1.** Seven PET-negative patients outside the study protocol were injected with standard 18F-FDG dose and acquired at 60 minutes post-injection. A subsequent second scan of the upper abdomen was obtained, using the same elapsed time used in the study protocol. By the comparison of the liver 18F-FDG uptake in the two scans, a correction factor of 1.063 was extracted and applied when comparing SUV values in PET-processed vs. PET-native images. Results are presented in Supplemental Table 1. **Supplemental Table 1.** Data for SUV correction factor. **Supplemental Table 2.** Study population.

## Data Availability

The datasets used during the current study are available from the corresponding author on reasonable request.

## Abbreviations

AAA: Advanced accelerator applications
AI: Artificial intelligence
EANM: European Association of Nuclear Medicine
EARL: EANM Research Ltd.
FDG: Fluorodeoxyglucose
GDPR: General data protection regulations
GMP: Good manufacturing practice
ICC: Intraclass correlation coefficients
OSEM: Ordered subsets expectation maximization
PACS: Picture Archiving and Communication System
PET/CT: Positron emission computed tomography
QC: Quality control
TOF: Time of flight
VOI: Volume of interest
