# Supplementary material for: Artificial intelligence for reduced dose 18F-FDG PET examinations: a real-world deployment through a standardized framework and business case assessment
Source: EJNMMI Phys. 2021 Mar 9;8:25. doi: 10.1186/s40658-021-00374-7 (PMC7943690; doi:10.1186/s40658-021-00374-7)
Supplement: Supplementary file 1 — Additional file 1: Appendix 1. Seven PET-negative patients outside the study protocol were injected with standard 18F-FDG dose and acquired at 60 minutes post-injection. A subsequent second scan of the upper abdomen was obtained, using the same elapsed time used in the study protocol. By the comparison of the liver 18F-FDG uptake in the two scans, a correction factor of 1.063 was extracted and applied when comparing SUV values in PET-processed vs. PET-native images. Results are presented in Supplemental Table 1. Supplemental Table 1. Data for SUV correction factor. Supplemental Table 2. Study population. [file 40658_2021_374_MOESM1_ESM.docx]

**Appendix 1**

Seven PET-negative patients outside the study protocol were injected with standard 18F-FDG dose and acquired at 60 minutes post-injection. A subsequent second scan of the upper abdomen was obtained, using the same elapsed time used in the study protocol. By the comparison of the liver 18F-FDG uptake in the two scans, a correction factor of 1.063 was extracted and applied when comparing SUV values in PET-processed vs. PET-native images. Results are presented in Supplemental Table 1.

**Supplemental Table 1.** Data for SUV correction factor

| Patients | PET-processed  (60 min) | PET-native  (late scan) |
| --- | --- | --- |
|  | *Liver SUVmean* | *Liver SUVmean* |
| Sample Pt-1 | 1.55 | 1.49 |
| Sample Pt-2 | 1.53 | 1.45 |
| Sample Pt-3 | 1.92 | 1.85 |
| Sample Pt-4 | 1.72 | 1.64 |
| Sample Pt-5 | 1.22 | 1.21 |
| Sample Pt-6 | 2.50 | 2.41 |
| Sample Pt-7 | 2.06 | 1.71 |
|  | **Mean Values** | |
|  | 1.786 | 1.680 |
|  | **Standard Deviation** | |
|  | ± 0.418 | ± 0.382 |
| Calculated correction factor= 1.063 | | |

**Supplemental Table 2.** Study population

| **ID** | **Sex** | **Age**  **(years)** | **Body Weight**  **(kg)** | **Injected Dose  (MBq)** | **Standard Dose (MBq)** | **PET Scanner** | **Disease** |
| --- | --- | --- | --- | --- | --- | --- | --- |
| Pt-1 | M | 77 | 72 | 141 | 210 | 1 | Rectal-Colon Carcinoma |
| Pt-2 | M | 62 | 75 | 143 | 215 | 2 | NHL |
| Pt-3 | F | 61 | 51 | 114 | 167 | 3 | Lung Cancer |
| Pt-4 | F | 61 | 83 | 170 | 250 | 2 | Lung Cancer |
| Pt-5 | M | 59 | 70 | 137 | 204 | 1 | HL |
| Pt-6 | M | 50 | 78 | 155 | 230 | 3 | Lung Cancer |
| Pt-7 | M | 79 | 80 | 156 | 240 | 1 | Lung Cancer |
| Pt-8 | F | 65 | 60 | 128 | 185 | 3 | Rectal-Colon Carcinoma |
| Pt-9 | M | 55 | 70 | 137 | 204 | 1 | HL |
| Pt-10 | M | 32 | 61 | 126 | 185 | 2 | Rectal-Colon Carcinoma |
| Pt-11 | F | 74 | 60 | 126 | 185 | 2 | NHL |
| Pt-12 | F | 78 | 54 | 114 | 167 | 1 | Lung Cancer |
| Pt-13 | F | 51 | 64 | 126 | 189 | 3 | Breast Cancer |
| Pt-14 | F | 73 | 66 | 132 | 196 | 1 | Pancreatic Cancer |
| Pt-15 | F | 72 | 60 | 129 | 185 | 1 | Gynaecological Cancer |
| Pt-16 | F | 68 | 65 | 133 | 192 | 2 | Lung Cancer |
| Pt-17 | F | 63 | 50 | 111 | 167 | 1 | NHL |
| Pt-18 | F | 61 | 69 | 134 | 204 | 3 | Breast Cancer |
| Pt-19 | M | 72 | 73 | 144 | 213 | 1 | Rectal-Colon Carcinoma |
| Pt-20 | F | 73 | 70 | 137 | 204 | 2 | Gynaecological Cancer |
| Pt-21 | M | 49 | 73 | 144 | 213 | 1 | Head and Neck Cancer |
| Pt-22 | F | 72 | 62 | 126 | 189 | 3 | Rectal-Colon Carcinoma |
| Pt-23 | M | 58 | 67 | 131 | 198 | 2 | Lung Cancer |
| Pt-24 | F | 65 | 40 | 100 | 148 | 3 | Gynaecological Cancer |
| Pt-25 | F | 59 | 70 | 136 | 204 | 3 | Breast Cancer |
| Pt-26 | M | 84 | 55 | 112 | 167 | 2 | Lung Cancer |
| Pt-27 | F | 35 | 51 | 113 | 167 | 3 | NHL |
| Pt-28 | M | 71 | 80 | 163 | 240 | 2 | Cholangiocarcinoma |
| Pt-29 | M | 72 | 65 | 128 | 192 | 3 | NHL |
| Pt-30 | F | 75 | 50 | 115 | 167 | 1 | Rectal-Colon Carcinoma |
| Pt-31 | F | 54 | 75 | 145 | 215 | 2 | Gynaecological Cancer |
| Pt-32 | M | 65 | 66 | 131 | 196 | 1 | Lung Cancer |
| Pt-33 | M | 77 | 72 | 140 | 210 | 1 | Rectal-Colon Carcinoma |
| Pt-34 | M | 74 | 68 | 130 | 196 | 2 | Lung Cancer |
| Pt-35 | M | 66 | 85 | 173 | 259 | 1 | Rectal-Colon Carcinoma |
| Pt-36 | F | 56 | 48 | 111 | 166 | 3 | Breast Cancer |
| Pt-37 | F | 75 | 40 | 99 | 148 | 1 | NHL |
| Pt-38 | F | 65 | 51 | 112 | 167 | 3 | Gynaecological Cancer |
| Pt-39 | M | 75 | 67 | 131 | 198 | 2 | NHL |
| Pt-40 | M | 74 | 90 | 185 | 277 | 1 | Lung Cancer |
| Pt-41 | F | 75 | 59 | 121 | 181 | 3 | Breast Cancer |
| Pt-42 | M | 51 | 69 | 135 | 200 | 2 | Lung Cancer |
| Pt-43 | M | 71 | 93 | 192 | 289 | 1 | Melanoma |
| Pt-44 | F | 55 | 86 | 176 | 262 | 1 | Melanoma |
| Pt-45 | M | 73 | 80 | 162 | 240 | 2 | Rectal-Colon Carcinoma |
| Pt-46 | F | 33 | 43 | 100 | 148 | 3 | Breast Cancer |
| Pt-47 | F | 56 | 82 | 167 | 252 | 2 | Rectal-Colon Carcinoma |
| Pt-48 | F | 71 | 65 | 128 | 192 | 2 | Gynaecological Cancer |
| Pt-49 | F | 47 | 100 | 199 | 296 | 3 | Gynaecological Cancer |
| Pt-50 | F | 68 | 55 | 111 | 167 | 3 | Gastric Cancer |
| Pt-51 | F | 57 | 88 | 181 | 270 | 2 | Breast Cancer |
| Pt-52 | M | 82 | 65 | 120 | 192 | 1 | Melanoma |
| Pt-53 | F | 64 | 58 | 117 | 178 | 3 | Rectal-Colon Carcinoma |
| Pt-54 | F | 57 | 58 | 119 | 178 | 2 | Breast Cancer |
| Pt-55 | F | 66 | 52 | 113 | 167 | 3 | Rectal-Colon Carcinoma |
| Pt-56 | F | 65 | 76 | 155 | 226 | 2 | Gastric Cancer |
| Pt-57 | M | 73 | 60 | 126 | 185 | 2 | Pancreatic Cancer |
| Pt-58 | F | 73 | 85 | 175 | 259 | 1 | Lung Cancer |
| Pt-59 | M | 69 | 95 | 199 | 296 | 2 | Rectal-Colon Carcinoma |
| Pt-60 | F | 74 | 72 | 141 | 210 | 3 | Gynaecological Cancer |
| Pt-61 | F | 72 | 44 | 100 | 148 | 3 | Pancreatic Cancer |
